# Delayed Pulmonary Apoptosis of Diet-Induced Obesity Mice following *Escherichia coli* Infection through the Mitochondrial Apoptotic Pathway

**DOI:** 10.1155/2019/1968539

**Published:** 2019-10-22

**Authors:** Fengyuan Wang, Zhicai Zuo, Zhuangzhi Yang, Kejie Chen, Jing Fang, Hengmin Cui, Gang Shu, Yi Zhou, Yi Geng, Ping Ouyang

**Affiliations:** ^1^College of Veterinary Medicine, Sichuan Agricultural University, Chengdu, Sichuan 611130, China; ^2^Chengdu Academy of Agriculture and Forestry Sciences, Chengdu, Sichuan 611130, China; ^3^School of Public Health, Chengdu Medical College, Chengdu, Sichuan 610500, China; ^4^College of Life Science, Sichuan Agricultural University, Ya'an, Sichuan 625014, China

## Abstract

*Escherichia coli* (*E. coli*) is one of pathogens causing nosocomial pneumonia and could induce pulmonary excessive apoptosis. Although much has been learned about metabolic diseases induced by obesity, the information linking bacterial pneumonia to obesity is limited. Accordingly, we investigated the apoptosis of normal (lean) and diet-induced obesity (DIO, fed a high-fat diet) mice after nasal instillation with *E. coli*. Lung tissues were obtained at 0 (preinfection), 12, 24, and 72 h after infection, and acute pulmonary inflammation was observed at 12 h. Elevated cell apoptosis and percentage of pulmonary cells depolarized with collapse of the mitochondrial transmembrane potential (*Δψ*m) occurred in response to bacterial infection. The relative mRNA and protein expressions of Bax, caspase-3, and caspase-9 increased, but Bcl-2 decreased in the lung. Interestingly, the apoptotic percentage and most of apoptosis-associated factors mentioned above peaked at 12 or 24 h in the lean-*E. coli* group, while at 24 or 72 h in the DIO-*E. coli* group. Taken together, these findings indicated that the *E. coli* pneumonia caused excessive pulmonary apoptosis through the mitochondria-mediated pathway, and the apoptosis was delayed in the DIO mice with *E. coli* pneumonia.

## 1. Introduction

Obesity has developed into a considerable health problem in the whole world. Obese people are under threat of respiratory symptoms, even with no obvious respiratory illness [1], and may have an increased risk of pneumonia [2]. The adverse effects of obesity on the respiratory system, like increasing airway resistance and the work of breathing, impairing respiratory muscle function and gas exchange [3], are mediated by a number of mechanisms, including production of proinflammatory cytokines by adipose tissue, mechanical restriction of thoracic volumes, and obesity-induced hypoventilation [4]. As mentioned above, obese individuals are more susceptible to pneumonia, but paradoxically, improved outcomes, like reduced mortality, are noticed in studies of acute bacterial pneumonia among obese ones [5–7].

Apoptosis has been recognized and accepted as a distinctive and important mode of “programmed” cell death [8]. As described in the literature, oxidative stress could cause cell apoptosis via both the mitochondria-dependent and mitochondria-independent pathways [9]. Reactive oxygen species (ROS), one of the most important products during oxidative stress, is a collective of oxygen-derived free radicals, which is produced by uncoupling, disturbance, or inhibition of mitochondrial respiratory chain. High ROS exposure gives rise to oxidative damage to mitochondrial DNA which consequently induces cell apoptosis [10]. Meanwhile, ROS is supposed to be involved in obesity [11]. A link between nutritional status and apoptosis reveals that high caloric intake may impair mitochondria for apoptosis [12]. Moreover, inflammation is a cellular response to stress, injury, or infection [13]. During infection, cells undergo apoptosis to inhibit the spread of microbes by directly killing or depriving the cellular resources for survival and replication [14, 15]. While screening these findings, although a new insight into the link between obesity and infection was provided, it is not clear whether cell apoptosis is involved.

We recently carried out experiments, in which diet-induced obesity (DIO) mice presented a delayed inflammatory response and oxidative stress in nonfatal acute pneumonia induced by *E. coli* infection [16]. It is well known that inflammation and oxidative stress can induce apoptosis in theory. Since there is little known about how pulmonary cell apoptosis impacted on the lungs of DIO mice following acute bacterial pneumonia, we purchased ICR mice fed high-fat diets, and then instilled the mice intranasally with *E. coli*, to shed light on the variations of pulmonary cell apoptosis between the normal and DIO mice following acute bacterial pneumonia.

## 2. Materials and Methods

### 2.1. Animal Model of Obesity

Three-week-old male ICR mice were purchased from Dossy Animal Center (Chengdu, China) and housed under specific pathogen-free conditions. All animal experimental procedures were approved according to the National and International Guidelines and by Sichuan Agricultural University Animal Care and Use Committee (Approval No. 2012-024).

Mice received either a normal diet or a high-fat diet, obtained from Dossy Animal Center according to our previous study [17]. The content of fat, mainly coming from the lard and soybean oil, was about 7% in the normal diet or 35.2% in the high-fat diet, respectively [18]. Food and water were supplied *ad libitum*. After feeding high-fat diets for 8 weeks, mice were weighed, and the ones whose obese index exceeded 20% were defined as successful obesity induction [19].

(1) $$\begin{array}{c} \text{Obese index}=\frac{\text{individual weight of DIO}-\text{average weight of lean}}{\text{average weight of lean}}\times 100\%. \end{array}$$

### 2.2. Groups and Pulmonary Infection Model

After feeding the high-fat diet for 8 weeks, the obese index of about 90% mice exceed 20%. Then, the mice fed either normal diets or high-fat diets were divided into 2 groups (144/group) as lean and DIO (diet-induced obese). The 2 groups were divided into 4 groups (72/group), namely, lean-*E. coli*, lean-uninfected, DIO-*E. coli*, and DIO-uninfected. Mice were sampled at 0 h (preinfection), 12 h, 24 h, and 72 h after *E. coli* infection.

*Escherichia coli* was obtained from the Veterinary Medical Laboratory of Sichuan Agricultural University (Ya'an, China), and the highest homologous with anthropogenic U00096 *E. coli* was cultured in Luria-Bertani broth at 37°C for 18 hours. Then, the bacterial culture was centrifuged, and bacterial pellets were resuspended in PBS to produce the inoculums. After being anesthetized with ether, mice in the lean-*E. coli* or DIO-*E. coli* group were instilled intranasally with 40 *μ*L inoculum of *E. coli* (containing approximately 4 × 10^9^ colony-forming units) suspended in phosphate-buffered saline (PBS) as reported previously [20]. And the same amount of PBS was given to the mice in the lean-uninfected or DIO-uninfected group by the same method.

### 2.3. Lung Injury Assayed by Histopathology

After infection with *E. coli* for 12 h, the lungs of eight mice from each group were immediately fixed in 4% paraformaldehyde and then dehydrated in alcohol, embedded by paraffin, sectioned at 5 *μ*m, and processed for hematoxylin and eosin staining. Histopathological changes were observed and photographed with a digital camera under 200x and 400x magnifications (Nikon DS-Ri1, Japan).

### 2.4. Measurement of Cell Apoptosis by Flow Cytometry

At indicated time point, the lungs from eight mice in each group were sampled and prepared into the single-cell suspension at a concentration of about 1 × 10^6^ cells/mL. After fluorescence staining by annexin V-fluorescein isothiocyanate (V-FITC) and propidium iodide (PI) at room temperature for 15 min in the dark, the cells were resuspended with annexin binding buffer, and the percentage of apoptotic cells was assayed by a flow cytometer (BD FACSCalibur) within 1 h. The annexin V-FITC kit was obtained from BD Pharmingen (559763, USA).

### 2.5. Measurement of Mitochondrial Transmembrane Potential (*Δψ*m)

0.5 mL single-cell suspension prepared above (containing about 5 × 10^5^ cells) was cultured with JC-1 working solution at 37°C for 20 min under a 5% CO_2_ incubator. After washing and suspending with JC-1 assay buffer, the mitochondrial membrane potential was assayed by a flow cytometer. The JC-1 kit was obtained from BD Pharmingen (USA, 551302).

### 2.6. Quantitative Real-Time PCR

At indicated time points, the lungs from eight mice in each group were crushed into powder with liquid nitrogen. Total RNA was prepared from TRIzol (9108/9109, Takara, Otsu, Japan) according to the manufacturer's recommendation, reverse transcribed with random hexamers (Prim-Script™ RT reagent Kit, RR047A, Takara, Japan), and amplified with specific primers. The primers were designed using Primer 5 software or NCBI primer pick online (Table 1) and synthesized at Sangon Biotech (Shanghai, China). The expression of caspase-3, caspase-9, Bax, and Bcl-2 transcript is shown relative to that of *β*-actin using the 2^-*ΔΔ*CT^ method.

### 2.7. Western Blotting

The tissue proteins were extracted with RIPA lysis buffer. After being equalized for total protein concentration, the protein was separated by SDS-PAGE and subjected to semidry blotting onto nitrocellulose membranes. The membrane was blocked and incubated overnight with rabbit anti-mouse caspase-3, caspase-9, Bax, Bcl-2, and GAPDH antibodies (ab32503, ab182858, ab184787, and ab202068, Abcam; 5174, Cell Signaling Technology) at 4°C. After being incubated with the peroxidase-conjugated goat anti-rabbit IgG (7074, Cell Signaling Technology), the blot was visualized by ECL™ (P0018A, Beyotime Technology) and X-ray film. Then, the expression of apoptosis-associated proteins is shown relative to that of GAPDH using Quantity One software.

### 2.8. Immunohistochemistry

The paraffin sections were treated with 3.0% hydrogen peroxide followed by boiling sodium citrate solution and incubated overnight with rabbit anti-mouse primary antibodies against caspase-3, caspase-9, Bax, and Bcl-2 at 4°C. Then, the sections were executed with SABC methods (SA1020, Wuhan Boster Bio-Engineering Limited Company, China) and visualized by DAB. Finally, the stained sections were photographed with a digital camera under 1000x magnification (Nikon DS-Ri1, Japan).

### 2.9. Statistical Analysis

The SPSS 17.0 statistical software package program for Windows was used for statistical tests. All results were expressed as the mean ± standard deviation. The significant differences among the four groups were analyzed by variance analyses (LSD or Dunnett's T3). A value of *p* < 0.05 was accepted as a statistically significant difference. The change rate was calculated by the following formula, and DIO and lean in the figures indicated the change rate of DIO and lean mice, respectively.

(2) $$\begin{array}{c} \text{Change rate }\left(\%\right)=\frac{\text{value of infected mice}-\text{value of uninfected mice }}{\text{value of uninfected mice}}\times 100\%. \end{array}$$

## 3. Results

### 3.1. Pathological Injuries of the Lung following *E. coli* Infection

**Figure 1:**
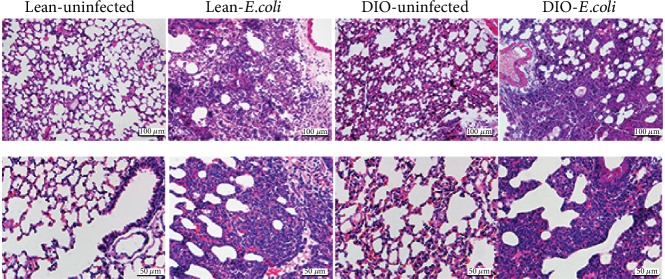
The representative histopathological changes of the lung at 12 h after infection. HE staining. Scale bars = 50 *μ*m (400x) or 100 *μ*m (200x).

As shown in Figure 1, the lung exhibited typical acute inflammation in either the lean- or DIO-*E. coli* group at 12 h after infection. Many neutrophils infiltrated into the bronchioles and alveolar lumen. Moreover, hyperaemia and hemorrhage of the alveolar wall were observed, as well as adjacent alveolar fusion and compensatory enlargement.

### 3.2. Changes in the Percentages of Apoptotic Cells in the Lung following *E. coli* Infection

**Figure 2:**
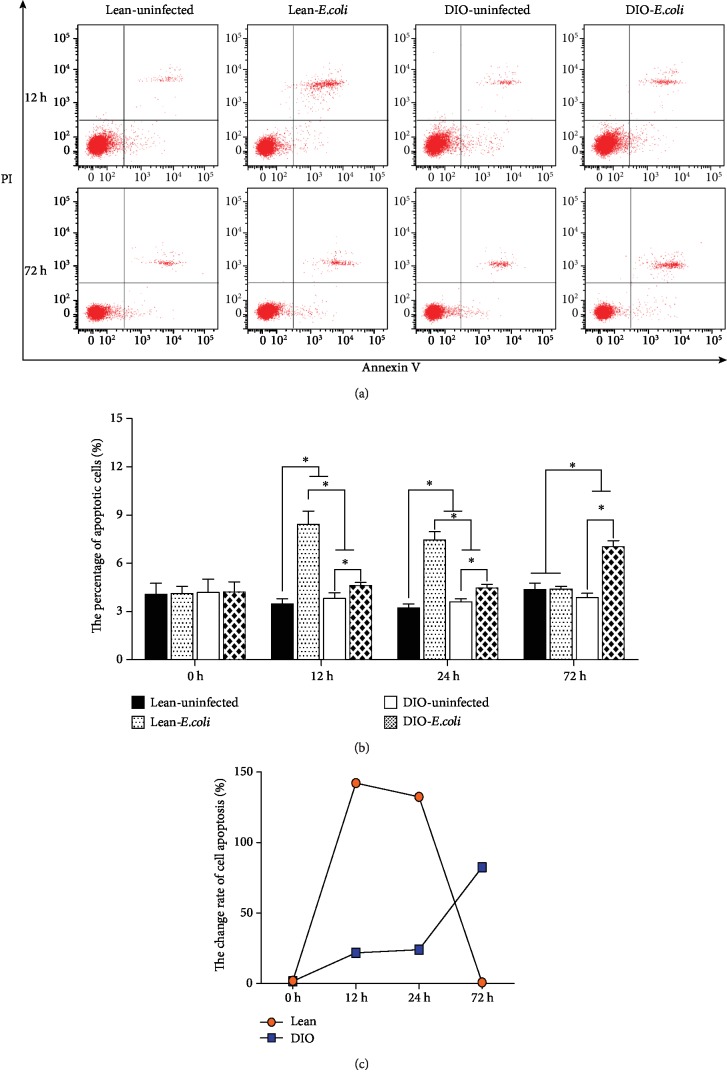
Apoptotic pulmonary cells. (a) Representative scattergrams of apoptotic pulmonary cells by flow cytometry at 12 h and 72 h. (b) The percentages of apoptotic pulmonary cells (%). (c) The change rates of pulmonary cell apoptosis (%). ^∗^The significant difference (*p* < 0.05).

As shown in Figure 2(a), cells in the left lower quadrant represent apoptotic negative cells, and cells in the right lower or upper quadrant represent apoptotic cells at an early phase or a late phase, respectively. The changes in the percentage of apoptotic cells in the lung displayed a different tendency between the lean and DIO groups (Figure 2(b)). The percentage of apoptotic cells in the lean-*E. coli* group was significantly higher (*p* < 0.05) than that in the lean group only at 12 h and 24 h, while the value in the DIO-*E. coli* group was significantly higher (*p* < 0.05) than that in the DIO-uninfected group from 12 h to 72 h. Moreover, the line chart (Figure 2(c)) showed that the change rate of apoptotic cell percentage in the lean mice peaked at 12 h, while that in the DIO mice continued to rise to 72 h.

### 3.3. Effect of Mitochondrial Transmembrane Potential (*Δψ*m) in the Lung following *E. coli* Infection

**Figure 3:**
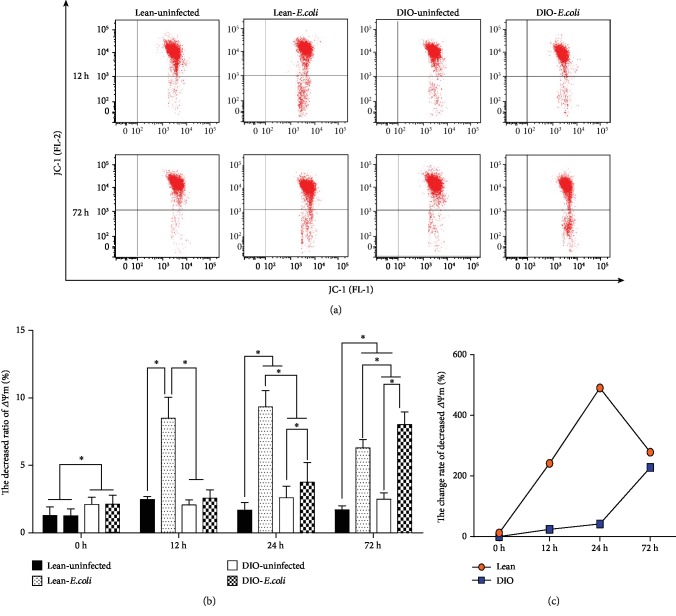
Pulmonary mitochondria transmembrane potential (Δ*ψ*m). (a) Representative scattergrams of reduced pulmonary Δ*ψ*m by flow cytometry at 12 h and 72 h. (b) The decreased ratios of pulmonary Δ*ψ*m (%). (c) The change rates of decreased pulmonary Δ*ψ*m (%). ^∗^The significant difference (*p* < 0.05).

The induction of apoptosis was associated with the perturbation of mitochondrial functions. Here, the changes in *Δψ*m were examined using fluorescent dye JC-1. Cells in the right upper quadrant represent high electronegativity, and cells in the right lower quadrant represent low electronegativity (Figure 3(a)). As shown in Figures 3(a) and 3(b), the percentages of pulmonary cells depolarized with collapse of *Δψ*m were significantly increased (*p* < 0.05) in the lean-*E. coli* group from 12 h to 72 h compared with the lean-uninfected group. However, the values in the DIO-*E. coli* group were higher only at 24 h and 72 h (*p* < 0.05) than those in the DIO-uninfected group. The change rate of decreased *Δψ*m of pulmonary cell was similar to the change rate of apoptotic percentage (Figure 3(c)).

### 3.4. Changes in Bax, Bcl-2, Caspase-3, and Caspase-9 Relative mRNA Expressions in the Lung following *E. coli* Infection

**Figure 4:**
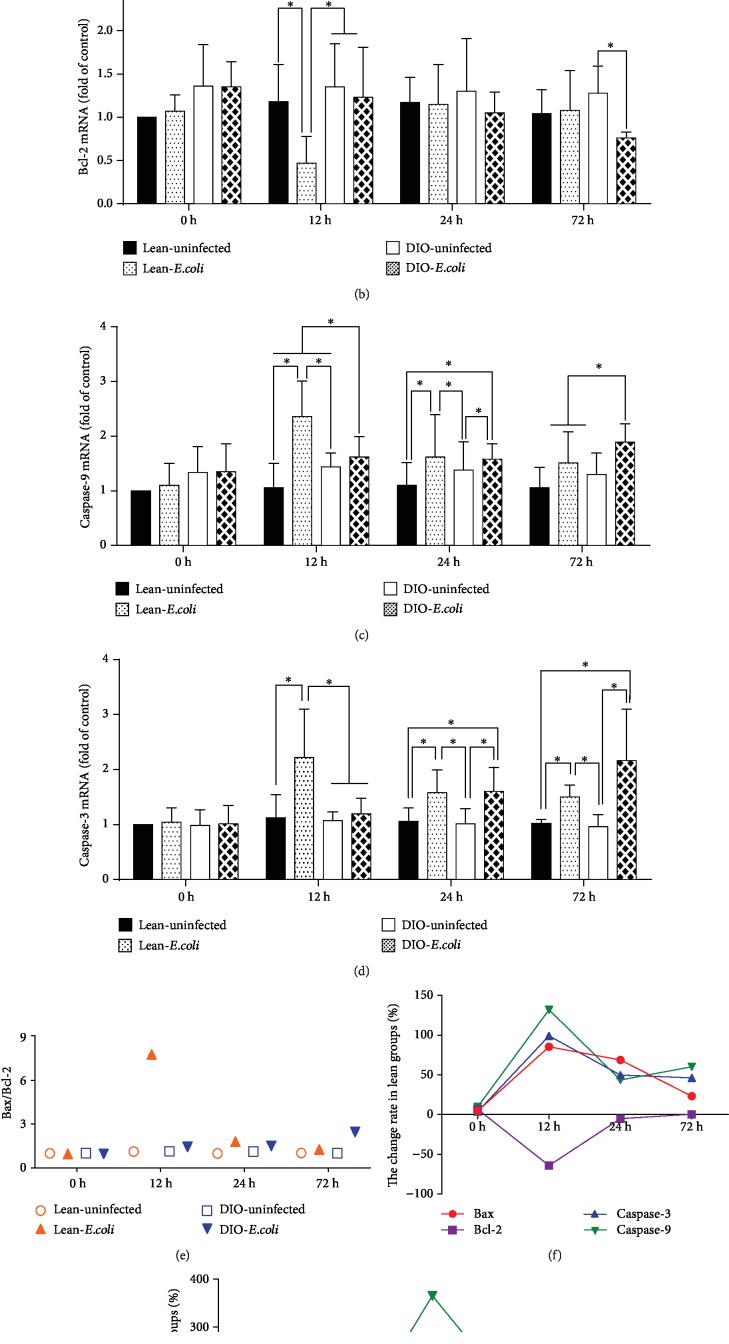
mRNA expressions of apoptotic factors associated with the mitochondrial pathway. (a–d) The mRNA levels of Bax, Bcl-2, caspase-3, and caspase-9 (fold of control). (e) The ratio of Bax/Bcl-2 mRNA. (f, g) The change rates of pulmonary apoptotic factor mRNA expression in the lean and DIO mice. ^∗^The significant difference (*p* < 0.05).

In the lean-*E. coli* group, the mRNA expression levels of Bax and caspase-9 were significantly increased (*p* < 0.05) at 12 h and 24 h and caspase-3 from 12 h to 72 h in comparison to the lean-uninfected group, while Bcl-2 was significantly decreased (*p* < 0.05) at 12 h. When compared with those of the DIO-uninfected group, the Bax mRNA levels of the DIO-*E. coli* group were all significantly increased at 72 h, as well as caspase-3 and caspase-9 at 24 h and 72 h (*p* < 0.05), while Bcl-2 was significantly decreased (*p* < 0.05) only at 72 h (Figures 4(a)–4(d)).

Among the four groups, the lean-*E. coli* group exhibited the highest ratio of Bax/Bcl-2 at 12 h but the DIO-*E. coli* group at 72 h (Figure 4(e)). As exhibited by the line chart (Figures 4(f) and 4(g)), the change rates of these apoptotic regulators were the highest in the lean mice at 12 h, while the peak change rates in the DIO mice were delayed to 24 h or 72 h.

### 3.5. Changes in Bax, Bcl-2, Caspase-3, and Caspase-9 Relative Protein Expression in the Lung following *E. coli* Infection

**Figure 5:**
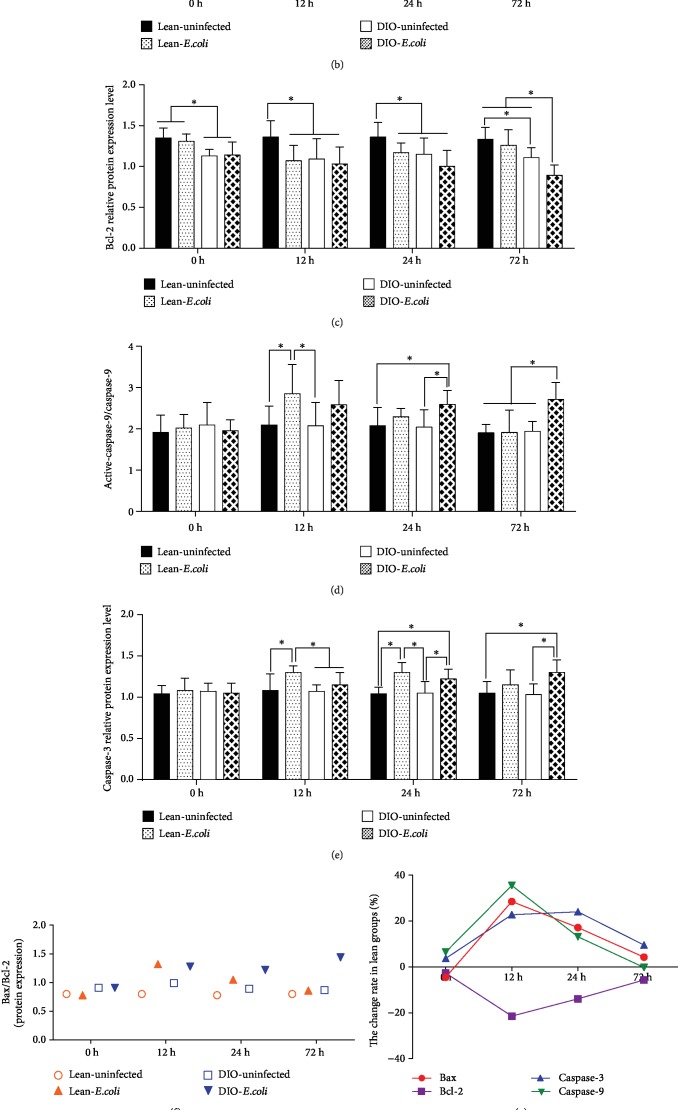
Protein expression of apoptotic factors associated with the mitochondrial pathway. (a) Representative western blot of protein expression. (b–e) The relative protein expression levels of Bax, Bcl-2, caspase-3, and caspase-9. (f) The ratio of Bax/Bcl-2 protein. (g, h) The change rate of pulmonary apoptotic factor protein expression in the lean and DIO mice. ^∗^The significant difference (*p* < 0.05).

As shown in Figure 5, the relative protein expressions of Bax and caspase-9 were significantly increased in the lean-*E. coli* group in comparison to the lean-uninfected group at 12 h (*p* < 0.05) and caspase-3 at 12 h and 24 h. Compared with the DIO-uninfected group, the caspase-3 and caspase-9 protein levels were significantly increased in the DIO-*E. coli* group at 24 h and 72 h (*p* < 0.05) and Bax at 12 h and 72 h (*p* < 0.05). Furthermore, the Bcl-2 protein level was lower in the DIO mice than in the lean mice at 0 h (*p* < 0.05). After infection, the Bcl-2 protein value declined at 12 h and 24 h in the lean-*E. coli* group and at 72 h in the DIO-*E. coli* group when compared with each uninfected control, respectively (*p* < 0.05).

The increased tendency of the Bax/Bcl-2 protein expression ratio was similar to its mRNA expression ratio (Figure 5(f)). Conclusively, the line chart of change rate showed that the apoptotic protein levels changed the most at 12 h in the lean mice but at 72 h in the DIO mice (Figures 5(g) and 5(h)).

### 3.6. Subcellular Localization of Bax, Bcl-2, Caspase-3, and Caspase-9 Proteins in the Lung

**Figure 6:**
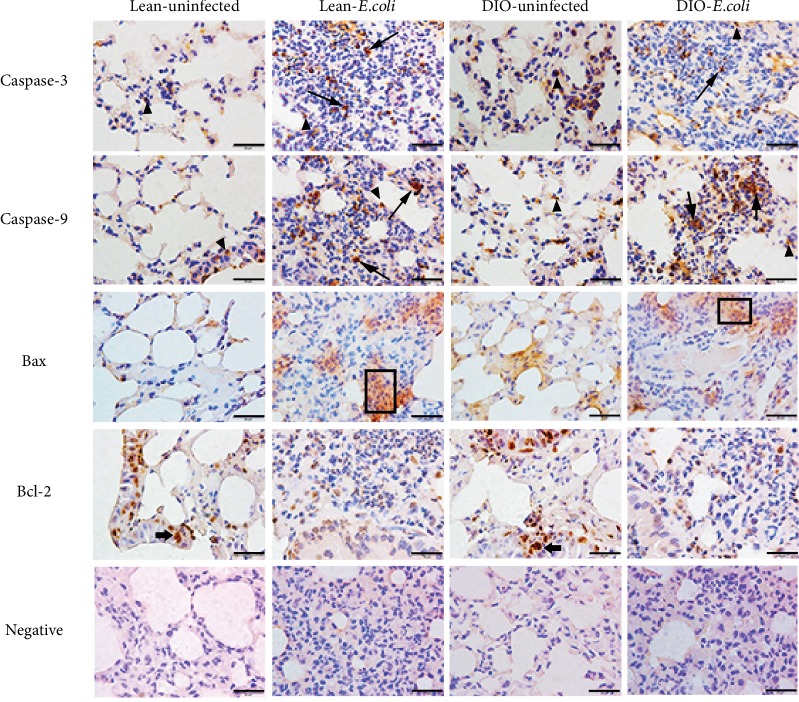
Representative immunohistochemistry staining of apoptotic proteins associated with the mitochondrial pathway at 12 h. DAB. Scale bar = 20 *μ*m (1000x). Positive caspase-3 or caspase-9 proteins in neutrophils (long arrows). Positive caspase-3 or caspase-9 proteins in alveolar epithelia (arrowheads). Positive Bax proteins in the neutrophil-infiltrated areas (boxes). Positive Bcl-2 proteins in the epithelial cells of respiratory bronchioles (broad arrows).

As shown in Figure 6, a few positive caspase-9 proteins were observed on the alveolar wall in the lean- and DIO-uninfected groups. After infection, large numbers of positive caspase-9 were visualized in the neutrophil-infiltrated areas or the alveolar wall in the lean- and DIO-*E. coli* groups. The location of caspase-3 protein was similar to that of caspase-9, but its content was lower than that of caspase-9. Bax-positive protein presented a scattered distribution, and a few Bax were seen in the alveolar wall of the uninfected groups, while there were more Bax in the neutrophil-infiltrated areas of the *E. coli-*infected groups. On the contrary, numerous Bcl-2-positive proteins appeared mainly in the epithelial cells of respiratory bronchioles in the uninfected groups but a few Bcl-2 in the *E. coli*-infected groups.

## 4. Discussion

*Escherichia coli* is one of the possible etiologies of nosocomial pneumonia, as well as a strong inducer of proinflammatory cytokine production from alveolar macrophages [21]. In the present study, 10^9^ CFUs/mL *E. coli* was intranasally instilled in mice (either lean or DIO) to establish acute pneumonia. According to histopathological observation, a typical acute inflammation appeared in the lung with a large number of neutrophils infiltrating into the alveolar and bronchiolar lumen. When the inflammation occurred, these inflammatory cells produced various cytokines. Thus, after infection, the cytokine and adipocytokine levels were significantly increased in mice [16].

In bacterial infection, the host is mainly dependent on the selective phagocytosis of neutrophils to eliminate invaders [22]. Neutrophils are able to synthesize and secrete proinflammatory cytokines in response to a variety of inflammatory stimuli [23]. And some typical cytokines, like tumor necrosis factor- (TNF-) *α*, interferon- (IFN-) *γ*, and interleukins (IL), can trigger cell apoptosis [24]. Moreover, neutrophil recruitment can activate the oxidative response, which is a primary host defense mechanism in acute pneumonia and a mediator of apoptosis [25, 26]. In addition, the generation of reactive oxygen species (ROS) in oxidative stress is capable to induce mitochondrial DNA damage and trigger apoptosis [27]. Our previous experiments indicated that pulmonary oxidative stress was notable in the mice after nasal instillation with *E. coli* [16]. Above all, acute bacterial infections were fairly associated with apoptosis, and this study puts emphasis on the mitochondrial apoptosis pathway between the lean and DIO mice with acute *E. coli* pneumonia.

As mentioned above, bacteria play an important role in triggering apoptosis. The mechanism of apoptosis in pulmonary diseases has two main hypotheses, namely, “neutrophilic hypothesis” and “epithelial hypothesis” [28], which means that cell apoptosis in pneumonia happened in two cell types, neutrophils and epithelia. Extensive evidences of neutrophil and alveolar epithelial cell apoptosis have been described on bacterial pneumonia and lipopolysaccharide- (LPS, one of the most important virulence factors of gram-negative bacteria) induced lung injury [29–31]. Besides, neutrophil regulates and alleviates inflammation through spontaneous apoptosis [32, 33]. In accordance with these researches, through flow cytometry, increased percentages of cell apoptosis were detected in the lean- and DIO-*E. coli* groups in comparison to the uninfected groups in the present study. For further study, four important factors involved in the mitochondrial-mediated apoptotic pathway were detected. In the mitochondrial pathway, antiapoptotic and proapoptotic proteins interact with the mitochondria and determine cell fate. The Bcl-2 family executes two opposing functions, including prosurvival proteins, such as Bcl-2, Bcl-w, and MCL-1, and proapoptotic proteins, such as Bax, Bid, and Bad [34]. These proteins regulate the release of cytochrome c from the mitochondrial innermembrane space, forming apoptosome with apoptotic protease-activating factor 1 (Apaf-1), activating caspase-9, thus initiating a caspase cascade which ultimately leads cell apoptosis [35, 36]. Researches on human monocytic U937 cells and epithelial HEp-2 cells showed that *E. coli* could induce apoptosis with an increased expression of Bax and a reduced expression of Bcl-2, which resulted in increased levels of released cytochrome c, caspase-3, and caspase-9 [37, 38]. In accordance with previous studies, after being infected with *E. coli*, the expressions of Bax, caspase-3, and caspase-9 were significantly increased while Bcl-2 was decreased in the infected groups.

Interestingly, the dramatic fold change in the percentage of cell apoptosis and the expression of apoptotic parameters were noted at 12 h or 24 h in the lean-*E. coli* group, whereas at 24 h or 72 h in the DIO-*E. coli* group. Meanwhile, the increased cytokine and adipocytokine levels were peaked at 12 h or 24 h in the lean mice, while these parameters continually increased along the infection time and peaked in the DIO mice at 72 h post infection [16]. These results indicated that the DIO mice may need longer time to respond to the inflammation than the lean mice. As it is well known, obesity is a medical condition, in which excess body fat increases body weight, resulting in more production of adipokines secreted by adipose tissue [39, 40]. Leptin is the first discovered adipokine derived from adipocyte and can modulate neutrophil chemotaxis and ROS release [41]. Previous studies have reported that leptin showed antiapoptotic properties on neutrophil via the NF-*κ*B and MEK1/2 MAPK pathways and led to delayed neutrophil apoptosis *in vitro* [42], inhibited thymic cells apoptosis through JAK-2 activation and IRS-1/PI3-K pathway in Wistar rats [43], and reduced degenerative nucleus pulposus cell apoptosis via promoting autophagy *in vitro* [44]. Other various adipokines, like vaspin, visfatin, and adiponectin, could inhibit apoptosis as well. Vaspin acts as a ligand for the cell-surface GRP78/VDAC complex inhibiting endothelial cell apoptosis. Visfatin shows antiapoptotic properties in TNF-*α*-induced apoptosis in breast cancer cells and palmitate-induced apoptosis in pancreatic *β*-cells [45, 46]. Adiponectin inhibits neutrophil apoptosis via activation of AMPK, PKB, ERK 1/2, and MAPK [47]. Therefore, obesity with increased levels of adipokines, like leptin and vaspin, might inhibit or delay cell apoptosis, which was accordance with our present results. Furthermore, cytokines and oxidative stress induced by inflammation are capable in triggering apoptosis [24, 27]. Following the infection, the proapoptotic effect of cytokines and oxidative stress was enhanced gradually, which counteracted the inhibited or delayed cell apoptotic effect executed by adipokines, resulting in a greater cell apoptotic rate in the DIO-*E. coli* group after 24 h. These results also exhibited a significant role for neutrophil apoptosis in inflammation. Indeed, we found that, through immunohistochemistry staining (especially caspase-3-positive proteins), two types of cells, neutrophils and epithelial cells, underwent apoptosis during infection, but more importantly, adipokines could inhibit neutrophil constitutive or spontaneous apoptosis. Thus, the delayed or inhibited neutrophil apoptosis by DIO during infection was partly able to determine the infection process in the lung.

The mitochondrion is a double-membrane-bound organelle found in most eukaryotic organisms and acts as a source of chemical energy (adenosine triphosphate (ATP)) supply in cells [48]. By stimuli, the mitochondrion-mediated apoptosis can be initiated in a receptor-independent manner that increases mitochondrial inner membrane permeability accompanied by *Δψ*m depolarization [49]. Stimulated by inflammation and infection, the proapoptosis protein bax and the antiapoptosis protein bcl-2 combined with ANT (adenine nucleotide translocator) or VDAC (voltage-dependent anion channel) competitively, and regulated the switch of the MPTP (mitochondria permeability transition pore). Once PT pores open, the mitochondrial transmembrane potential would dramatically decrease, leading the release of cytochrome c and activating caspase-9 gradually. Obese individuals have reduced oxidative phosphorylation (OXPHOS) gene expression and oxygen consumption and increased oxidative stress and ROS production, causing mitochondrial dysfunction [50]. In the present study, the percentage of pulmonary cells depolarized with the collapse of the *Δψ*m was higher in the DIO mice than in the lean mice. On the contrary, obese Zucker rats displayed no difference in oxygen consumption, ATP synthesis, membrane potential, citrate synthase, and cytochrome c oxidase activities compared with lean Zucker rats [51]. After infection, the percentage significantly increased in both the lean- and DIO-*E. coli* groups, and the increase was more dramatic at 12 and 24 h in the lean-*E. coli* group but at 72 h in the DIO-*E. coli* group. These results suggested that *E. coli* pneumonia caused the Δ*ψ*m change, by which the mitochondrion-mediated apoptotic pathway was activated in the lung of both the lean and DIO mice, but delayed in the latter.

For subcellular localization of these apoptotic factors, immunohistochemistry was performed. Bax should be in the membrane of mitochondria [52]. In the present duty, Bax-positive protein was detected as dispersive distribution in the lean- and DIO-*E. coli* groups. Identical to a previous report, Bcl-2 immunostaining is cytoplasmic and granular and restricted in normal bronchial epithelium to the basal epithelial layer or to some epithelial cells that are perpendicularly oriented to the basal lamina before infection [53], whereas, after infection, the Bcl-2 protein expression in the bronchial epithelial cells almost vanished and only a few Bcl-2 were noted in the neutrophil-infiltrated areas. Caspase-3 and caspase-9 were located in the mitochondria, cytosol, and nucleus in cells [54, 55]. After being infected with *E. coli*, caspase-3 and caspase-9 proteins were mainly displayed in the cytoplasm of inflammatory cells and sloughed pulmonary epithelial cells in the neutrophil-infiltrated areas. Taken together, immunohistochemistry results suggested that these mitochondrion-mediated apoptotic proteins were mainly located in the neutrophil-infiltrated areas after infection.

## 5. Conclusions

In conclusion, nasal infection with *E. coli* was able to establish bacterial pneumonia in mice. And after being infected with *E. coli*, both the lean and DIO mice exhibited increased percentages of apoptosis; decreased pulmonary *Δψ*m; upregulated expressions of Bax, caspase-3, and caspase-9 mRNA and protein; and downregulated expression of Bcl-2. However, most impressively, almost all the above-mentioned parameters peaked at 12 h or 24 h in the lean-*E. coli* group but at 24 h or 72 h in the DIO-*E. coli* group. These results indicated that the DIO mice presented a delayed cell apoptosis in the acute pneumonia induced by *E. coli* infection through the mitochondrial apoptotic pathway. Meanwhile, the major cell exhibiting delayed apoptosis by obesity might be neutrophils in the mice with *E. coli* pneumonia. The observations reported here provide the foundation for further investigations on the relationship between obesity and bacterial infection.

## Figures and Tables

**Table 1:** Sequence of primers used in qRT-PCR.

Target gene	Accession number	Primer sequence (5′-3′)	Product size
Caspase­3	NM_009810.3	Forward: ACATGGGAGCAAGTCAGTGG Reverse: CGTCCACATCCGTACCAGAG	149 bp
Caspase­9	NM_015733.5	Forward: GAGGTGAAGAACGACCTGAC Reverse: AGAGGATGACCACCACAAAG	103 bp
Bax	NM_007527.3	Forward: ATGCGTCCACCAAGAAGC Reverse: CAGTTGAAGTTGCCATCAGC	163 bp
Bcl-2	NM_009741.5	Forward: AGCCTGAGAGCAACCCAAT Reverse: AGCGACGAGAGAAGTCATCC	159 bp
*β*-Actin	NM_007393	Forward: GCTGTGCTATGTTGCTCTAG Reverse: CGCTCGTTGCCAATAGTG	117 bp

## Data Availability

The cytokine contents and oxidative stress data used to support the findings of this study have been deposited in the PubMed repository (10.1038/s41598-018-32420-3). The flow cytometry, qRT-PCR, and western bolt data used to support the findings of this study are included within the article.
